# Identification of Proteins and MicroRNAs with Prognostic Value for Assisted Reproduction Technology Outcomes in Follicular Fluid of Women with Endometriosis: A Pilot Study

**DOI:** 10.3390/ijms26199752

**Published:** 2025-10-07

**Authors:** Ana Catarina Neto, Cláudia Freitas, Ângela Ribeiro, Adriana R. Rodrigues, João L. Silva-Carvalho, Henrique Almeida, Delminda Neves

**Affiliations:** 1 Departmento de Biomedicina—Unidade de Biologia Experimental, Faculdade de Medicina da Universidade do Porto, 4200-319 Porto, Portugal; caty.neto@gmail.com (A.C.N.); angela.ribeiro@ceti.pt (Â.R.); adrod@med.up.pt (A.R.R.); almeidah@med.up.pt (H.A.); 2 Instituto de Investigação e Inovação em Saúde da Universidade do Porto (i3S), 4200-135 Porto, Portugal; 3 Reproductive Medicine, Obstetrics and Gynaecology Department, Hospital Dr. Nélio Mendonça, Serviço de Saúde da Região Autónoma da Madeira (SESARAM), 9004-514 Funchal, Portugal; freitascv@gmail.com; 4 CETI—Centro de Estudo e Tratamento da Infertilidade, 4100-118 Porto, Portugal; jlsilvacarvalho@gmail.com; 5 Faculdade de Nutrição e Alimentação da Universidade do Porto, 4150-180 Porto, Portugal; 6 Obstetrics and Gynaecology, Hospital CUF Porto, 4100-180 Porto, Portugal

**Keywords:** anti-Mullerian hormone, assisted reproductive techniques, biochemical pregnancy, bone morphogenetic protein-15, endometriosis, growth differentiation factor-9, human follicular fluid, infertility, microRNAs

## Abstract

This study aims to identify molecular markers with prognostic value for biochemical pregnancy in follicular fluid (FF) samples from women with endometriosis after assisted reproductive technology (ART) intervention. Levels of growth differentiation factor-9 (GDF-9), bone morphogenetic protein-15 (BMP-15), and anti-Mullerian hormone (AMH) proteins were semi-quantified by Western blotting and microRNAs 20a_1, 145_1, 320a_1, 125-b-5p, 212-3p, and 199_a by qPCR in FF samples from women submitted to ART with a previous diagnosis of endometriosis (n = 20) or male factor infertility (controls) (n = 44). An increase in GDF-9 and BMP-15 and a decrease in AMH mature protein were observed, as well as an increase in miR20a_1 (*p* = 0.04), miR145_1 (*p* = 0.003), and miR320a_1 (*p* = 0.006) in FF samples collected from women with endometriosis compared with controls. A reduction was observed in miR125b-5p (*p* = 0.004) and 212-3p (*p* = 0.02) in endometriosis. Receiver operating characteristic (ROC) curve analysis indicated that miR125b-5p, miR212-3p, and miR-145_1 are potential predictors of endometriosis, and miR145_1 and miR320a_1 of biochemical pregnancy in controls. Although limited by a small sample size, the current study demonstrated alterations in AMH, BMP-15, GDF-9, and specific miRNA levels in FF samples harvested from women with endometriosis, emphasizing their potential roles in endometriosis-related infertility. These microRNAs, dysregulated in women with endometriosis, unveil their biomarker properties and their predictive value for ART success.

## 1. Introduction

Infertility affects approximately 15% of couples in Western societies [1]. Among the female causes of infertility, endometriosis presents a major contribution, considering that it globally afflicts 5–15% of women of reproductive age and up to 40% of infertile women [2].

Endometriosis is characterized by ectopic presence of endometrial-like glands and stroma that cause pelvic pain and anatomic distortion [3]. The pathophysiology of the disease remains poorly understood, but it is known that endometriosis comprises a complex inflammatory response induced by pro-oxidative conditions, cellular proliferation, migration and invasion, and angiogenesis [4,5].

Patients with endometriosis-associated infertility often require assisted reproductive technology (ART) treatments [6,7]. During oocyte harvesting for in vitro fertilization or intra-cytoplasmatic sperm injection, follicular fluid (FF) is aspirated and usually discarded. Because FF composition parallels composition of the serum as a result of molecular diffusion, it presents a high potential for use in the detection of markers with prognostic value, despite rarely being analyzed in the setting of ART treatments. Indeed, FF contains a multitude of hormones, metabolites, polysaccharides, growth factors [8], and microRNAs (miRNAs/miRs) (small, non-coding RNAs approximately 22–25 nucleotides in length) [9,10], mostly secreted by theca and granulosa cells (GCs), that reflect metabolic activity and follicular development phase, influence oocyte quality and success of fertilization, and ultimately embryo development [11].

Growth differentiation factor-9 (GDF-9) and bone morphogenetic protein-15 (BMP-15) are two oocyte-secreted factors (OSFs) that exhibit greater homology between themselves than with the remaining members of the transforming growth factor β (TGF-β) family [12]. Those OSFs appear to intervene synergistically in cellular mechanisms and are crucial for folliculogenesis and female fertility [13]. However, while GDF-9 silencing in mice results in decreased GC proliferation, abnormal oocyte growth, and follicular development failure [14] because GDF-9 inhibits follicular atresia and GC apoptosis [15], BMP-15 induces proliferation of those cells [16].

In contrast to GDF-9 and BMP-15, anti-Mullerian hormone (AMH), also a member of the TGF-β family, is produced by GCs from 36 weeks of gestation until menopause and considered a marker of oocyte reserve [17,18]. In endometriosis patients, AMH levels are diminished in peritoneal fluid, even when serum levels are normal, suggesting that AMH may have a role in the disease [19]. The discrepancy between circulating and local levels suggest differential regulation of AMH synthesis or disposal in endometriosis. In this setting, miRNAs are potential contributors on account of their recognized role as post-transcriptional regulators of gene expression [20]. MicroRNAs are concentrated in exosomes and secreted into body fluids, such as blood and FF, which makes them putative prognostic biomarkers of ART outcome in endometriosis, owing to their easy accessibility and stability under experimental conditions [10,21,22]. Several miRNAs have been implicated in endometriosis-related infertility (Table 1). In particular, miR-125b-5p is upregulated in serum and plasma of women with endometriosis, being considered a putative marker of endometriosis [10] and contributing to systemic inflammation and potentially altering the follicular and oocyte environment through PI3K/Akt (phosphatidylinositol 3-kinase (PI3K)/protein kinase B) and MAPK (mitogen-activated protein kinase) pathways [23,24]. In FF, miR-212-3p and miR-320a have been associated with embryo developmental potential and oocyte and embryo quality, respectively [25,26,27]. These miRs correlate with fertilization success and embryo quality in in vitro fertilization (IVF) [25], processes that may be compromised in endometriosis [28], with lower levels of both linked to impaired fertilization outcomes [26,29]. miR199a, miR20a, and miR145 are involved in TGF-β signalling [30,31,32], impacting crucial endometriosis-associated mechanisms, such as hypoxia, angiogenesis, inflammation, and cell proliferation [33,34]. While miR-20a is frequently dysregulated in endometriosis tissue and serum, which links to disease recurrence, implantation failure, follicular dysfunction, angiogenesis, and cell proliferation [35,36,37], miR-145 has been shown to suppress GC proliferation in mice [38]. Functionally, overexpression of miR-145 in human endometriotic cell lines (12Z) inhibits proliferation, invasion, and stem-like traits by repressing pluripotency factors and cytoskeletal proteins expression [39]. Moreover, miR-199a levels are also altered in the serum of patients, with lower levels linked to disease severity according to the endometriosis fertility index (EFI) and poorer fertility outcomes, possibly due to miR-199a impact in extracellular matrix remodelling, inflammation, and lesion invasiveness [40,41,42,43].

To identify biomarkers with prognostic value for ART outcomes in women with endometriosis, AMH, GDF-9, BMP-15, and miRNAs 20a_1, 145_1, 320a_1, 125-b-5p, 212-3p, and 199_a, were quantified in FF samples harvested from endometriosis patients that undertook ART procedures [27].

## 2. Results

### 2.1. Population Characteristics

Data relative to women enrolled in this study are summarized in Table 2. The average age of women from whom FF samples were collected, which impacts the expression of AMH and the quality of oocytes, was equivalent between groups: both in those employed for protein analysis (35 and 36.1 years in male factor and endometriosis groups, respectively, *p* = 0.5) and for miRNAs analysis (34.8 and 34.6 years in male factor and endometriosis groups, respectively, *p* = 0.8). As well, no differences were observed in body mass index or parity between women included in the two groups (*p* = 0.34). Neither the mean number of type A embryos observed at day 3 (*p* = 0.19), nor the percentage of transfers at day 3 or day 5, differ between patients with endometriosis and controls.

### 2.2. AMH Decreases and GDF-9 and BMP-15 Increases in FF Samples of Women with Endometriosis

**Figure 1:**
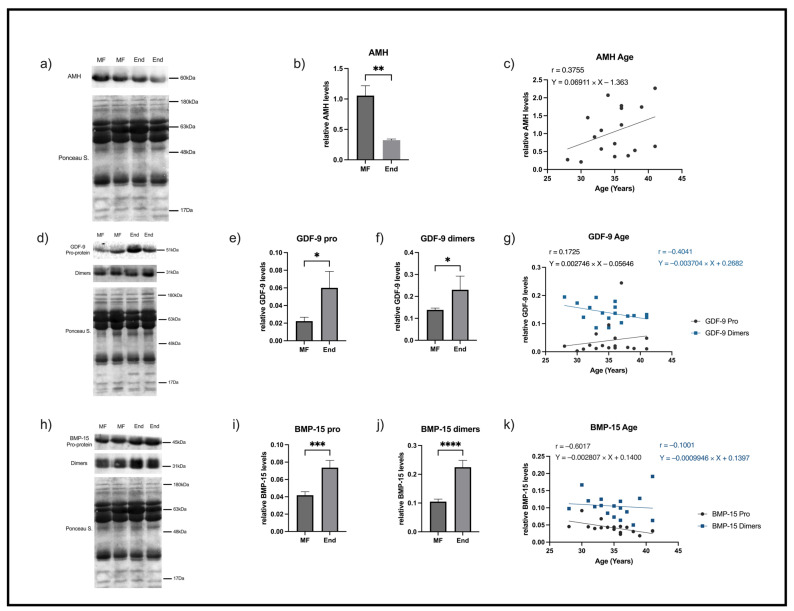
Representative blots and graphical depictions of AMH, GDF-9, and BMP-15 analyses in FF samples from 2 different patients of male factor group (MF) and 2 different patients with endometriosis (End). (**a**) A single band corresponding to AMH was detected with a molecular weight of 60 kDa. (**b**) AMH levels in MF/controls and endometriosis. (**c**) The correlation between AMH levels and age in controls. (**d**) Representative blots of GDF-9 pro-protein and homo/heterodimers in FF that show, respectively, a 51 KDa pro-protein single band (**e**) or 31 KDa dimers (**f**) in controls and endometriosis. (**g**) Representation of correlation between GDF-9 and age in controls. (**h**) Representative blots of FF BMP-15 pro-protein and homo/heterodimers showing, respectively, a single band with an apparent molecular weight of 45 kDa (**i**) and 31 KDa dimers (**j**) in controls and End. (**k**) Graphical representation of the correlation between BMP-15 levels and age in controls. For loading control of immunoblots the respective Ponceau S-stained membrane was used. AMH—anti Mullerian hormone, BMP-15—bone morphogenetic protein 15, End—endometriosis, FF—follicular fluid, GDF-9—growth differentiation factor 9, MF—male factor/controls (n = 17); endometriosis patients (End, n = 7). * *p* < 0.05 compared to MF, ** *p* < 0.01 compared to MF. *** *p* < 0.001 compared with MF, **** *p* < 0.0001 compared with MF.

A single band with an apparent molecular weight of 60 kDa corresponding to AMH protein was identified in all samples from both groups of women (Figure 1a). A decrease in AMH levels in FF samples of endometriosis patients was found relative to the controls (*p* = 0.009) (Figure 1b).

No correlation of AMH levels in FF with age was found within the group of women without disease through the Pearson correlation test [45] (Figure 1c).

GDF-9 was detected in all the studied FF samples, and three bands with different molecular weight were identified: the pro-protein (51 kDa) (Figure 1d) and the mature protein present in homodimeric/heterodimeric forms presenting two bands with very close molecular weight (about 31 kDa) (Figure 1d). An increase in expression of both the pro-protein (Figure 1e) and dimeric forms (Figure 1f) of GDF-9 in patients with endometriosis relative to the controls was found (*p* = 0.01 and *p* = 0.036, respectively). No Pearson correlation for GDF-9 levels and age [45] in the male factor group of women was found (Figure 1g).

BMP-15 was also identified by Western blotting, and, similarly to what was observed for GDF-9, three bands were detected in all FF samples. A pro-protein (45 kDa) (Figure 1h) more expressed in the group of patients with endometriosis was identified (*p* = 0.0009) (Figure 1i), and in line with this the dual band referring to protein present in homodimeric and heterodimeric complexes (about 31 kDa) (Figure 1j) was also significantly increased in this group (*p* < 0.0001) (Figure 1j). A negative correlation between the pro-form of BMP-15 levels and age (*p* = 0.01) in male factor group of women was found (Figure 1k). No correlation was found for BMP-15 dimer levels and age in those women.

### 2.3. Levels of miR125b-5p and miR212-3p Decrease and of miR20a_1, miR145_1, and miR320a_1 Increase in FF Samples of Women with Endometriosis

**Figure 2:**
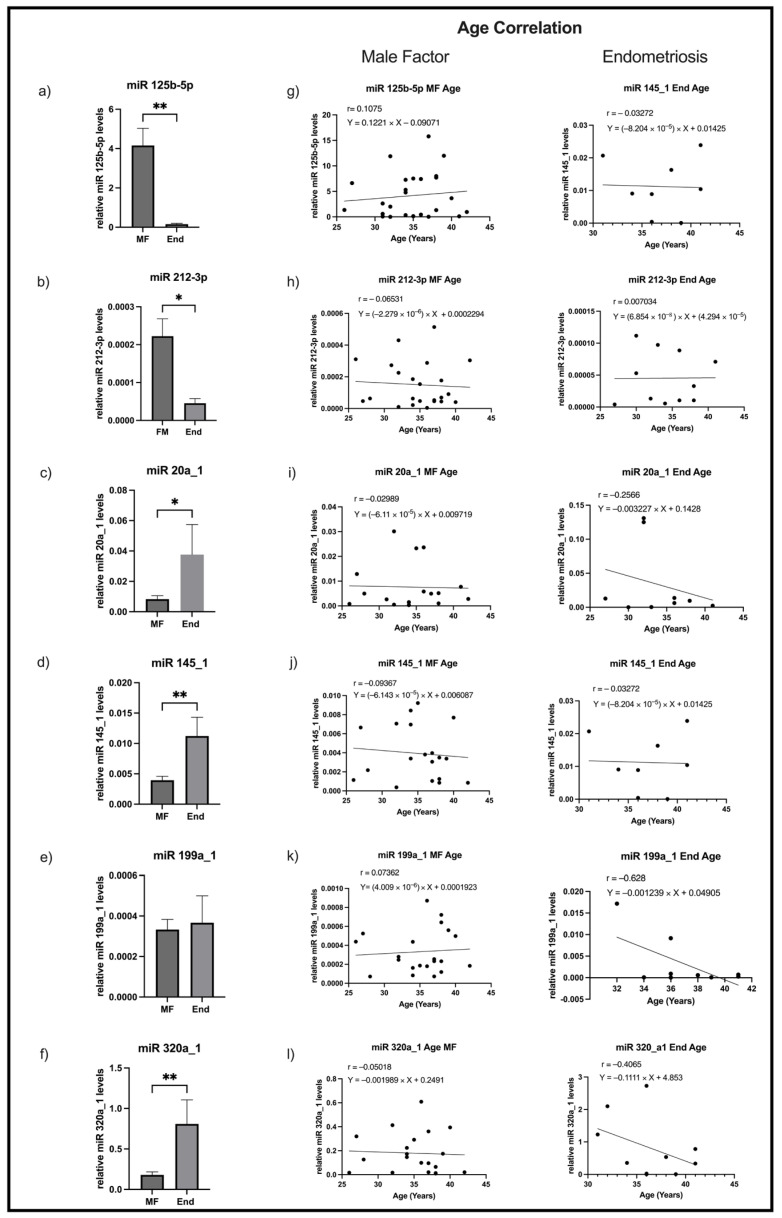
MiRNAs analyses. Graphical representation of expression levels of (**a**) miR125b-5p, (**b**) miR212-3p, (**c**) miR20a_1, (**d**) miR145_1, (**e**) miR199a_1, and (**f**) miR320a_1 assessed by RT-qPCR in FF samples of women with male factor/controls infertility (MF) and with endometriosis (End). (**g**–**l**) Graphical representation of correlation between miRs levels and age in male factor/controls (n = 32) and endometriosis women (n = 20). * *p* < 0.05 compared to MF, ** *p* < 0.01 compared to MF. End—endometriosis, MF—male factor.

The levels of miR125b-5p, miR212-3p, miR20a_1, miR145_1, miR199a_1, and miR320a_1 assessed by RT-qPCR are depicted in Figure 2.

Levels of miR125b-5p (Figure 2a) and miR212-3p (Figure 2b) were decreased in the FF samples of women with endometriosis when compared with controls (*p* = 0.004 and *p* = 0.017, respectively). On the other hand, levels of miR20a_1 (Figure 2c), miR145_1 (Figure 2d), and miR320a_1 (Figure 2f) were increased in the FF samples of women with endometriosis relative to those without disease (*p* = 0.04, *p* = 0.003 and *p* = 0.006, respectively). No difference was found in miR199a_1 level between the groups (Figure 2e).

To identify whether women’s age interferes in the FF levels of miRs, a Pearson correlation between the studied miRNAs and age in patients and controls was carried out separately for each miRNA (Figure 2g–l). Among those analyses, no correlations were found in neither endometriosis patients nor controls.

### 2.4. MiR125b-5p, miR212-3p, and miR145_1 Levels in FF Are Putatively Predictors of Endometriosis and miR145_1 and miR320a_1 of Biochemical Pregnancy

**Figure 3:**
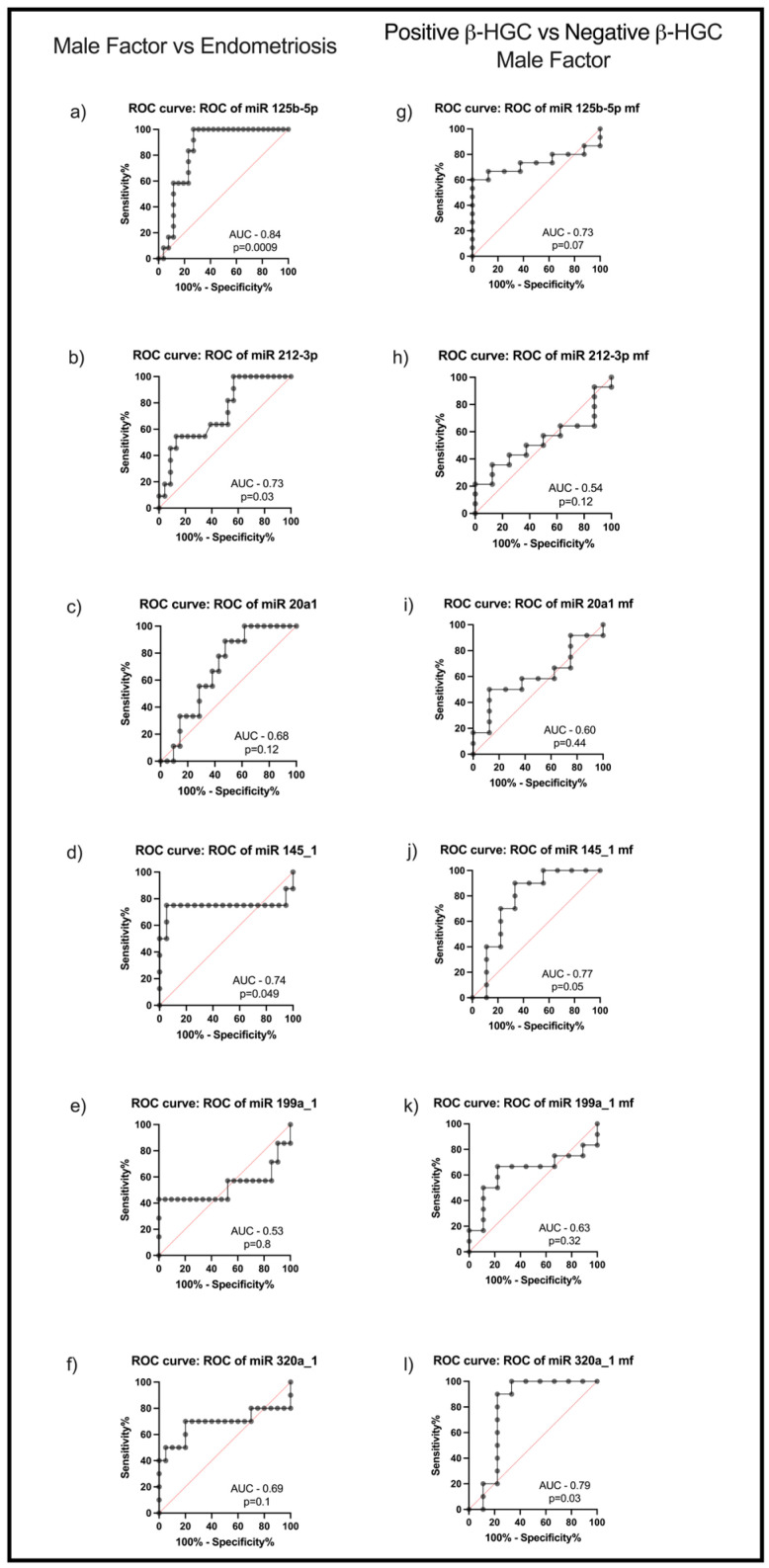
ROC analysis for miRNAs based on the real-time qPCR data. Male factor/controls vs. endometriosis for (**a**) miR125b-5p, (**b**) miR212-3p, (**c**) miR20a_1, (**d**) miR145_1, (**e**) miR199a_1, and (**f**) miR320a_1. Positive β-hCG vs. negative β-hCG in male factor group (**g**–**l**). AUC—area under the curve; β-hCG—human chorionic gonadotropin, ROC—receiver operating characteristic.

To assess whether the studied miRNAs could be valuable markers of endometriosis, ROC analysis was performed. Among the studied miRNAs, miR125b-5p (area under the curve—AUC: 0.84; *p* = 0.0009) (Figure 3a), miR-212-3p (AUC: 0.73; *p* = 0.03) (Figure 3b), and miR-145_1 (AUC: 0.74; *p* = 0.049) (Figure 3d) were shown to be putative predictors of endometriosis.

In addition, ROC analysis was also employed to verify whether studied miRNAs could be used as predictors for biochemical pregnancy achievement (β-human chorionic gonadotropin—hCG-positive test). In the control group of women, both miR-145_1 (AUC: 0.77; *p* = 0.05) (Figure 3j) and miR-320a_1 (AUC: 0.79; *p* = 0.03) (Figure 3l) levels in FF were shown to be indicators of increased probability of biochemical pregnancy achievement. Regarding miR125b-5p levels in FF (AUC: 0.73; *p* = 0.07) (Figure 3g), a strong parallel tendency was observed; however, it did not reach statistical significance.

## 3. Discussion

Infertility is a public health problem that leads to ART treatments for a growing number of couples. Research on the molecular mechanisms that underlie infertility has gained increasing importance, and analysis of biological samples, collected when oocytes are retrieved for ART interventions, has been identified as an important approach to predict the success of treatment [46,47]. In particular, FF is a valuable source for studying infertility causes since FF directly contacts and receives the molecules secreted by the oocyte, thus reflecting the microenvironment of the female gametes in ovaries. In the present study, the levels of AMH, GDF-9, BMP-15, and specific miRNAS in FF samples collected from patients with endometriosis were assessed in an attempt to identify molecular markers with prognostic value for biochemical pregnancy.

Our findings show that FF of women with endometriosis presented lower levels of AMH when compared with controls, indicating reduced ovarian reserve and supporting the impaired fertility often observed in endometriosis patients [17]. This result aligns with previous studies showing parallel decrease in AMH levels in FF and serum in women with endometriosis [48,49]. Additionally, Kitajima et al. reported a reduction in AMH levels in the peritoneal fluid, but not in serum, of women with endometriosis, further suggesting a local, pelvic, effect of AMH decline in a reduced fertility disease [19].

The decrease in AMH correlates with the increase in miRs targeting its mRNA, as reported by Belguith et al. who detected an upregulation of miR199a in the plasma of infertile women with dysregulated AMH levels [50]. We did not observe such a difference in miR199a levels in FF samples of endometriosis patients (possibly due to the different sample types here analyzed), but we found an increase in miR20a_1 and miR145_1, which have been reported as intervenients in AMH gene expression regulation through TGF-β signalling modulation [31,32,51,52].

The proteins GDF-9 and BMP-15, primarily secreted by the oocyte [53], are crucial for follicular development and oocyte quality [11,54]. Both proteins belong to the TGF-β family and form homo- and heterodimers [55], detectable in the FF [27,53]. In cumulus oophorus cells, when acting as homodimers or as heterodimers (known as cumulin) [56], they enhance oocyte maturation in vitro and improve embryo development and fetal survival in several animal models [56,57,58,59,60,61]. Levels of the monomeric form of GDF-9 protein, albeit rarely detected in FF, positively correlate with oocyte maturation and embryo development [27,62]. As well, high BMP-15 levels in FF, particularly in younger women, improve pregnancy chances [62,63]. In agreement with this observation, in the current study FF BMP-15 pro-protein levels in older women were lower when compared to younger women and with all the other studied peptides. This odd finding provides additional support to the view that BMP-15 has a major role in the follicle development of mono-ovulatory species such as humans [64,65] and sheep [66], in contrast to poli-ovulatory species [67], and it is interesting to verify that cumulus cell receptors for BMP-15 in humans show age-related decrements too [68].

In this study, an increase in BMP-15 and GDF-9 levels was found in FF samples of patients with endometriosis, both in pro-proteins and mature proteins in dimers, considering the 31kDa double band identified in blots when BMP-15 or GDF-9 were detected. That increase appears amidst conflicting GDF-9 results which show a decrease or no difference when women with severe endometriosis are compared to women without the disease [69,70,71,72]. The isolated assessment of GDF-9 pro-protein, compared to the simultaneous immature and mature protein assay of the present study, may underlie such differences. In addition, this discrepancy may also be attributed to the type of analysis, as some results were obtained from serum or granulosa cells, while others reflect mRNA expression rather than protein levels [70,72]. In contrast to GDF-9, BMP-15 levels agree with a previous observation [71], which provides additional consistency to BMP-15 relevance in human follicle growth and development. As a whole, the consistent increase in both GDF-9 and BMP-15 proteins, and the probable formation of homo- and heterodimers found in FF samples of women with endometriosis, favour their role as an intrinsic compensatory mechanism to enhance oocyte quality.

The analysis of miRNA levels in FF provides further insight into the molecular landscape of endometriosis. We found a decrease in miR125b-5p levels in FF of patients with endometriosis that contrasts with previous reports showing its upregulation in serum [40]. MiR125b-5p acts as a tumour suppressor, inhibiting cell proliferation and promoting apoptosis [73,74,75], processes that are dysregulated in endometriosis. Indeed, the decrease in miR-125b-5p in FF locally favours the inflammatory and proliferative nature of endometriosis [76]. Interestingly, the inflammatory response modulator miR212-3p [77,78] is also downregulated, which aligns with the disrupted immune environment in endometriosis. The potential contribution of miR125b-5p and miR212-3p to endometriosis establishment is supported by their significant predictive value for endometriosis found by ROC analysis, anticipating the clinical utility of both miRNAs as diagnostic tools. Besides miR125b-5p and miR212-3p, miR145_1 levels in FF also showed predictive value for endometriosis, distinguishing women with endometriosis-related infertility from fertile women undergoing ART due to male factors, which agrees with previous studies that highlight the role of specific miRNAs in endometriosis diagnosis and pathogenesis [37,79,80]. In addition, levels of miR145_1 and of miR320a_1 were found to be strong predictors of biochemical pregnancy success in women with male factor infertility, reinforcing that miRNAs in FF not only influence endometriosis, but may also impact fertility outcomes. Unfortunately, information relative to biochemical pregnancy outcomes in women with endometriosis is scarce, which impedes us concluding whether those miRs present equivalent prognostic value in those patients and constitutes a limitation of this study.

Levels of miR20a_1, miR145_1, and miR320a_1 were increased in FF of women with endometriosis. According to Andreas et al., the overexpression of miR20a_1, differently expressed in cumulus cells (higher) and oocyte (lower), accelerated maturation of oocytes in vitro [81]. MiR20a_1 directly targets phosphatase and tensin homologue (PTEN) and bone morphogenetic protein receptor type 2 (BMPR2) to regulate their expression both at the mRNA and protein levels in cultured GCs [82] and takes part in TGF-β signalling [31]. In fact, overexpression of both miR20a_1 and miR145_1 [32,51] inhibits TGF-β signalling and supresses cell migration and invasion [83]. Moreover, in line with our results, miR320_a levels were significantly upregulated in serum exosomes from patients with endometriosis [44]. This miRNA presents tumour suppressor properties [84,85,86] and modulates migration of endometrial stromal cells [87], which might result in a balanced condition when confronted with miR-125. It is interesting to note that miR320a_1 in FF had been associated with high-quality embryos [29], a finding consistent with the correlation we found for miR320a_1 levels in FFs of control women and biochemical pregnancy success. Together with data relative to GDF-9 and BMP-15, the increase in miR20a_1, miR145_1, and miR320a_1 levels in FFs of women with endometriosis supports an adaptive response to increase oocyte quality and mitigate endometriosis progression in patients.

Considering that pregnancy outcome in ART strongly depends on women age, Pearson’s correlation between levels of every analyzed molecule in FF and age were assessed. In what concerns OSFs, only a negative correlation for BMP-15 pro-protein and age was found, which agrees with the overall oocyte quality decrement along time [88]. We failed to identify a correlation between miRNA levels and women’s age, although previous studies evidenced variation in specific miRNAs with age in serum and in airways [89,90]. Although miRNAs are considered key regulators of reproductive processes [91], we hypothesize that miRNA levels in FF are more likely to be influenced by disease state than by patient age, reinforcing their potential as disease biomarkers.

However, the present study presents limitations. The main is the low sample size, particularly of women with diagnosed endometriosis. In addition, considering that FF analysis is restricted to women that are undertaking ART interventions, it will be of interest to assess levels of miRNAs with potential prognostic value in blood.

## 4. Materials and Methods

### 4.1. Follicular Fluid Collection and Processing

FF samples obtained from women aged 26–41 years submitted for ART after informed consent from 2008 to 2020 were assigned by CETI (Centro de Estudo e Tratamento de Infertilidade), Porto. The use of biological samples discarded during processing in ART was approved by the local Ethics Committee (Unidade de Bioética, Biodireito e Economia da Saúde DCSS—Faculdade de Medicina da Universidade do Porto). Rules and directives of the Helsinki Declaration were fully adopted. The harvest of the FF was conducted during the follicular puncture with a 17-gauge needle (Vitrolife, Göteborg, Sweden) on the largest follicle (diameter greater than 17mm, which predicts higher oocyte maturation and competence—both of which may serve as indirect indicators of oocyte quality) [92] identified by ultrasound at the most reachable ovary in each patient.

For this study, only samples from women who completed their ART treatment were selected. Completion of the treatment cycle was defined as undergoing embryo transfer, regardless of whether it was performed on the 3rd or 5th day of embryo development. The decision regarding the day for embryo transfer was based on the number of oocytes retrieved, fertilization rate, and the number of grade A embryos at the cleavage stage on days 2 and 3 of development. Embryo classification during the cleavage stage was based on three main morphological criteria: cleavage symmetry, blastomere size uniformity, and the extent of cytoplasmic fragmentation [93]. For instance, an embryo at approximately 68 h post-fertilization (corresponding to day 3 of development) is expected to present around eight blastomeres. Ideally, these cells should be symmetrical. Additionally, high-quality embryos (classified as Grade A) typically exhibit ≤25% cytoplasmic fragmentation. These characteristics are indicative of optimal developmental. In certain cases, previous endometrial maturation assessments and hormonal analyses on the day of the trigger for oocyte pick-up were performed to facilitate not only the decision on the transfer day but also on whether to proceed with a freeze-all strategy. The therapeutic indications and the execution of the most appropriate technique, either conventional IVF or intracytoplasmic sperm injection (ICSI), were followed to complete the respective cycle of treatment. Following the retrieval of cumulus-oocyte complexes, the FF of each woman were collected into conical-bottom tubes and centrifuged at 200× *g* for 6 min. After centrifugation, the supernatant was collected, aliquoted into 1 mL fractions, and stored at −20 °C for subsequent analysis.

Immediately after ovarian follicular puncture, FF samples were anonymized and classified according to the age of the patient and clinical diagnosis. According to infertility origin, FF samples were divided into two groups: male factor (control group) (n = 44) and endometriosis (n = 20). Data relative to women enrolled in this study are summarized in Table 2. All patients with infertility were evaluated for suspected endometriosis, starting with a clinical history and physical examination, followed by imaging techniques, such as ultrasound and, when indicated, magnetic resonance imaging. Laparoscopy was performed in patients presenting with symptoms strongly suggestive of endometriosis. In our dataset, we included patients with a history of previous surgery, those who underwent adapted hormonal suppression prior to ovarian stimulation, or both. Patients who presented co-morbidities, such as diabetes, arterial hypertension, asthma, depression, dyslipidemia, polycystic ovary syndrome, tubal issues, or endometriosis associated with other infertility cause, were excluded from the study. All the patients included in the control group were evaluated by a gynecologist, independently of the identification of male cause of infertility, to confirm the unlikeliness of endometriosis or exclude additional causes of infertility.

### 4.2. Semi-Quantification of AMH, BMP-15, and GDF-9 in FF by Western Blotting

Analysis of proteins in FF requires the prior removal of albumin, with the ALBUMINOUT^TM^ kit (G Biosciences, St. Louis, MO, USA) according to the manufacturer’s instructions. After this procedure, total protein in samples was quantified by the colorimetric method of Bradford [94]. Then, electrophoretic separation of 15 µg of total protein of each FF sample loaded on a 12% sodium dodecylsulfate–polyacrylamide resolution gel, was performed in a discontinuous buffer system (Biorad Laboratories, Hercules, CA, USA) [95] for approximately 1h at room temperature under a constant electric current of 25mA per gel.

After electrophoretic separation, proteins were transferred to a nitrocellulose membrane 0.45 μm (Biorad Laboratories), applying a potential difference of 30V for 90 min. After staining with Ponceau S for 2 min, membrane images were captured in Chemidoc TM XRS equipment (BioRad Laboratories). Then, membranes were incubated for 1h at room temperature with blocking solution (5% *w*/*v* non-fat powdered milk Molico^®^ in TBS with 0.1% *v*/*v* Tween-20 (TBS.T)), or alternatively 5% *w*/*v* bovine serum albumin (BSA) in TBS.T for membranes used for the detection of BMP-15, and then with primary antibodies, rabbit anti-AMH (Abcam, Cambridge, UK) diluted 1: 7500 *v*/*v* or goat anti-BMP-15 (R & D, Minneapolis, MN, USA) diluted 1: 1000 *v*/*v* in blocking solution, overnight at 4 °C and with stirring. GDF-9 was detected with a rabbit anti-GDF-9 antibody (Abcam) diluted 1:500 *v*/*v* or 1:250 *v*/*v* (for hetero- or homodimers, respectively) in blocking solution for two nights at 4 °C. Membranes were then washed with TBS.T and incubated with the appropriate secondary antibody conjugated with horseradish peroxidase diluted in blocking solution for 1h at room temperature with stirring. Immunoreaction of the membrane was performed through application of chemiluminescent substrate for peroxidase Clarity Western ECL SUBS (BioRad Laboratories) for 5 min, followed by signal detection in Chemidoc TM XRS equipment (BioRad Laboratories). Normalization of the studied proteins was carried out with the corresponding lane stained with Ponceau S. Each experiment was repeated three times.

### 4.3. Reverse Transcription qPCR

MiRNAs were extracted from FF samples using the miRNeasy Mini Kit (Qiagen, Hilden, Germany), according to the manufacturer’s instructions. Purified RNAs were converted to cDNA using miRCURY LNA RT kit (Qiagen). Then, miRNAs were analyzed in 384-well plates after mixing 1 mL of cDNA with 1 mL of miRNA-specific primer (miScript Primer Assay/miRCURY LNA miRNA assay) (Table 3), 3.5 mL of 2x miRCURY SYBR Green Master mix (Qiagen), and 4.5 mL of MQ water. Real-time polymerase chain reaction (qPCR) was conducted on a CFX Opus 384 real-time PCR system (BioRad Laboratories) and started with denaturation (10 min) at 95 °C, followed by 50 cycles of 15 s denaturation at 95 °C, 30 s annealing at 55 °C, and 30 s extension at 60 °C. PCR data were normalized with a control, RNU5G, using the formula 2^−(CT target–CT control)^ to quantify miRNA’s expression levels. RNU5G, a small nucleolar RNA, was selected as the reference gene for miRNA normalization due to its stability, abundance, size similarity, and comparable biological and technical properties to target miRNAs, as well as its high conservation across species. One negative control per experiment was prepared by omission of reverse transcriptase.

### 4.4. Pregnancy Assessment

All participants were instructed to undergo biochemical pregnancy assessment 12 days post-embryo transfer. This involved the collection of peripheral blood for the quantification of β-hCG levels by quimioluminesce analysis. In cases of a positive β-hCG result, clinical pregnancy was confirmed approximately 15 days later via transvaginal ultrasound.

All data were systematically recorded by associating the respective sample code with the corresponding pregnancy follow-up outcomes.

### 4.5. Statistical Analysis

Statistical analysis was performed using GraphPad Prism version 10.3.1 (GraphPad Software Inc. San Diego, CA, USA). Results are presented as mean ± standard error of the mean (SEM). Comparative analysis of the proteins studied was carried out by Student’s t test. The analysis of miRNAs was performed using a receiver operating characteristic (ROC) curve. Probability values of *p* < 0.05 were considered statistically significant. For the correlation analysis of the studied protein levels the Pearson correlation test was employed [45]. Considering that this is an exploratory study carried out in a small group of women, in which data are collected with an objective but not with a prespecified key hypothesis, multiple test adjustments, such as Bonferroni correction, were not performed [96].

## 5. Conclusions

In sum, our pilot study reveals significant alterations in AMH, BMP-15, GDF-9, and specific miRNAs in FF samples from women with endometriosis. While the increase in BMP-15 and GDF-9 may represent a compensatory mechanism to increase oocyte quality, miRNA dysregulation highlights their role as biomarkers and potential therapeutic targets in both endometriosis and ART management. These findings set the stage for further investigations into the molecular pathways influencing fertility in women with endometriosis.

## Figures and Tables

**Table 1:** MiRNA specific roles on endometriosis-related infertility.

miRNA	Role in Endometriosis-Related Infertility	Reference(s)
miR-125b-5p	Upregulated in endometriosis. Implicated in lesion invasion, inflammation, and alterations of the oocyte/follicular environment. Pathways: PI3K/Akt, MAPK, and inflammation-related signalling.	[10,23,24,40]
miR-212-3p	Higher levels are associated with improved embryo development. Pathways: Regulation of folliculogenesis and oocyte competence (specific targets not consistently identified).	[25,26]
miR-320a_1	Upregulated in serum exosomes of endometriosis patients. Associated with oocyte and embryo quality and IVF outcomes. Lower or altered expression linked to poorer embryo development. Pathways: Regulation of energy metabolism, oxidative stress, and embryo development genes.	[26,29,44]
miR-199a_1	Upregulated in serum of endometriosis patients. Considered in the EFI and related to disease severity. Lower levels correlate with worse EFI and poor fertility prognosis. Pathways: ECM remodelling, inflammation, and lesion invasiveness.	[40,41,42]
miR-20a_1	Dysregulated in endometriosis. Associated with disease recurrence and lesion biology. Influences implantation and follicular function, impacting fertility. Pathways: TGF-β signalling, cell proliferation, and angiogenesis.	[35,36,37]
miR-145_1	Downregulated in endometriosis (serum and lesions). Functional studies show regulation of granulosa cell proliferation. Impacts follicular cell behaviour, potentially reducing oocyte quality and ovarian function. Pathways: Cell-cycle regulation and expression of cytoskeletal-related genes.	[38,39]

Abbreviations: PI3K/Akt—phosphatidylinositol 3-kinase (PI3K)/protein kinase B; MAPK—mitogen-activated protein kinase; IVF—in vitro fertilization; EFI—endometriosis fertility index; ECM—extracellular matrix; TGF-β—transforming growth factor β.

**Table 2:** Resume of the characteristics of the women enrolled in the study.

	Age (Years)	Body Mass Index (Kg/m^2^)	Parity (Number of Live Births/% of Women)	Type A embryos at 3rd day (Average Number)	3rd Day Transfer (% of Cases)	5th Day Transfer (% of Cases)
Male factor (n = 34)	26–42	23.5	0/88 1/8 2/4	1.81	47.1	52.9
Endometriosis (n = 15)	27–41	21.9	0/67 1/33	2.63	46.7	53.3

**Table 3:** List of primers used in miRNAs quantification by reverse transcription qPCR.

MiRNA	Reference
RNU5G	hsa_miR-RNU5G miRCURY LNA miRNA PCR Assay (YP00203908)
miR-125b-5p	hsa_miR-125b-5p miRCURY LNA miRNA PCR Assay (YP00205713)
miR-212-3p_1	Mm_miR-212-3p_1 miScript Primer Assay (MS00024570)
miR-20a_1	Hs_miR-20a_1 miScript Primer Assay (MS00003199)
miR-199a_1	Hs_miR-199a_1 miScript Primer Assay (MS0006741)
miR-145_1	Hs_miR-145_1 miScript Primer Assay (MS00003528)
miR-320a_1	Hs_miR-320a_1 miScript Primer Assay (MS00014707)

## Data Availability

The data is not publicly available due to privacy and ethical restrictions but are available from the corresponding author by reasonable request.

## Abbreviations

The following abbreviations are used in this manuscript:

AUC	Area Under the Curve
AMH	Anti-Mullerian hormone
ART	Assisted Reproductive Technology
BMP-15	Bone morphogenetic protein-15
BSA	Bovine Serum Albumin
CETI	Centro de Estudo e Tratamento de Infertilidade
END	Endometriosis
FF	Follicular fluid
GC	Granulosa cells
GDF-9	Growth differentiation factor-9
β-hCG	Human Chorionic Gonadotropin
ICSI	Intracytoplasmic Sperm Injection
IVF	In vitro fertilization
MF	Male Factor
miR	MicroRNA
MiRNA	MicroRNA
OSFs	Oocyte-Secreted Factors
ROC	Receiver Operating Characteristic
TBS	Tris-buffer Saline
TGF-β	Transforming Growth Factor β
